# Brain regions associated with the acquisition of conditioned place preference for cocaine vs. social interaction

**DOI:** 10.3389/fnbeh.2012.00063

**Published:** 2012-09-24

**Authors:** Rana El Rawas, Sabine Klement, Kai K. Kummer, Michael Fritz, Georg Dechant, Alois Saria, Gerald Zernig

**Affiliations:** ^1^Experimental Psychiatry Unit, Medical University InnsbruckInnsbruck, Austria; ^2^Faculty of Health Science, Department of Clinical and Experimental Medicine, Linköping UniversitySweden; ^3^Institute for Neuroscience, Medical University InnsbruckInnsbruck, Austria

**Keywords:** conditioned place preference, drug abuse, social interaction, cocaine, reward, Zif268 expression, reinstatement, acquisition

## Abstract

Positive social interaction could play an essential role in switching the preference of the substance dependent individual away from drug related activities. We have previously shown that conditioned place preference (CPP) for cocaine at the dose of 15 mg/kg and CPP for four 15-min episodes of social interaction were equally strong when rats were concurrently conditioned for place preference by pairing cocaine with one compartment and social interaction with the other. The aim of the present study was to investigate the differential activation of brain regions related to the reward circuitry after acquisition/expression of cocaine CPP or social interaction CPP. Our findings indicate that cocaine CPP and social interaction CPP activated almost the same brain regions. However, the granular insular cortex and the dorsal part of the agranular insular cortex were more activated after cocaine CPP, whereas the prelimbic cortex and the core subregion of the nucleus accumbens were more activated after social interaction CPP. These results suggest that the insular cortex appears to be potently activated after drug conditioning learning while activation of the prelimbic cortex—nucleus accumbens core projection seems to be preferentially involved in the conditioning to non-drug stimuli such as social interaction.

## Introduction

Switching the preference of the substance dependent individual toward non-drug related activities remains one of the great challenges that confront drug dependence therapy. We have previously shown that only four social interaction episodes (15 min each) with a male early-adult conspecific as an alternative (i.e., non-drug-associated) stimulus completely reversed conditioned place preference (CPP) for cocaine (15 mg/kg i.p.) and were even able to inhibit cocaine-induced reinstatement of cocaine CPP (Fritz et al., 2011a). These protective effects of social interaction were paralleled by effects on the brain circuitry known to be involved in drug reinforcement and reward. Social interaction during extinction of cocaine CPP reversed cocaine CPP-reinstatement-associated Zif268 expression in the nucleus accumbens shell, the central and basolateral amygdala, and the ventral tegmental area (Fritz et al., 2011a). Furthermore, social interaction during extinction of cocaine CPP also reduced cocaine-CPP-stimulated FosB expression in the nucleus accumbens shell and core; and increased pCREB (cAMP response element binding protein) expression in the nucleus accumbens shell and the cingulate cortex area 1 (Cg1) (El Rawas et al., 2012). In addition, we have also shown that the sigma1 receptor antagonist BD1047 enhances reversal of CPP from cocaine to social interaction (Fritz et al., 2011b). These findings suggest that social interaction, if offered in a context that is clearly distinct from the previously drug-associated one, may profoundly decrease the incentive salience of drug-associated contextual stimuli. Touch (taction) appears to be the major rewarding sensory component of the composite stimulus “social interaction” (Kummer et al., 2011; Peartree et al., 2012).

If rats were concurrently conditioned for place preference by pairing cocaine with one compartment and social interaction with the other (i.e., mutually exclusive stimulus presentation during training), both stimuli (15 min dyadic social interaction vs. 15 mg/kg i.p. cocaine) produced equally strong CPP (Fritz et al., 2011a,c). Recently, we have shown that pre-acquisition lesioning the accumbens core or the basolateral amygdala shifted the animals' preference toward social interaction CPP, whereas a bilateral shell lesion shifted the preference toward cocaine CPP (Fritz et al., 2011c). These findings suggest a role of the nucleus accumbens shell in mediating alternative non-drug-associated conditioned stimuli (social interaction) and a role of the nucleus accumbens core and the basolateral amygdala in mediating drug (cocaine) associated conditioned stimuli (Fritz et al., 2011c). Previous pharmacological and lesion studies have identified several elements of the brain circuitry that mediate drug-seeking behavior. This brain circuitry has been shown to involve the basolateral (Kruzich and See, 2001; Fuchs et al., 2002, 2005; McLaughlin and See, 2003; Di Ciano and Everitt, 2004) and the central amygdala (Kruzich and See, 2001), the core of the nucleus accumbens (Di Ciano and Everitt, 2004; Fuchs et al., 2004; Ito et al., 2004; Kalivas and O'Brien, 2008), the dorsolateral caudate-putamen (Fuchs et al., 2006), the hippocampus (Fuchs et al., 2005), the dorsomedial prefrontal cortex (McLaughlin and See, 2003; Fuchs et al., 2005), the orbitofrontal cortex (Lasseter et al., 2009), the insula (Di Pietro et al., 2006; Kufahl et al., 2009) and the ventral tegmental area (McFarland and Kalivas, 2001). Furthermore, Kalivas and colleagues have reported that the rat prelimbic prefrontal cortex and nucleus accumbens core are critical for initiating cocaine seeking (Kalivas and O'Brien, 2008) while the infralimbic cortex and nucleus accumbens shell are recruited by extinction training to suppress cocaine seeking (Peters et al., 2008).

Our aim was to investigate the differential activation of brain regions involved in drug-seeking circuitry after acquisition of cocaine CPP or social interaction (non-drug) CPP. Therefore, we investigated Zif268 expression using immunohistochemistry in brain regions of (1) naive “untreated” rats, (2) rats that expressed cocaine CPP and (3) rats that expressed social interaction CPP. In order to investigate if brain activation patterns differ between the acquisition of either stimulus by naive animals and the reinstatement of a previously acquired cocaine preference in animals that had—or had not—a history of social interaction (Fritz et al., 2011a), we added Zif268 expression in brain regions of (4) rats that expressed cocaine CPP, then underwent extinction with saline in absence of social interaction and finally a reconditioning with cocaine in the previously cocaine-associated chamber and (5) rats that expressed cocaine CPP, then underwent extinction with saline and social interaction and finally a reconditioning with cocaine in the previously cocaine-associated chamber. We hypothesized that separate neural substrates mediate acquisition/expression of cocaine CPP vs. social interaction CPP. In the present study, we showed that neural substrates that mediate cocaine CPP and social interaction CPP are mostly overlapping. However, the granular insular cortex and the dorsal part of the agranular insular cortex were more activated after cocaine CPP, whereas the prelimbic cortex and the core subregion of the nucleus accumbens were more activated after social interaction CPP. Further work is necessary to characterize whether activating neurotransmitters or inhibiting neurotransmitters are released.

## Materials and methods

### Animals

Male Sprague Dawley rats weighing 150–250 g, corresponding to an age of (6–8 weeks) which can be considered early adulthood (Spear, 2000), were obtained from the Research Institute of Laboratory Animal Breeding of the Medical University Vienna (Himberg, Austria) and were group-housed (six rats per cage) at 24°C. The animals received *ad libitum* access to tap water and pellet chow. A 12-h light/dark cycle, with lights on from 0800 h to 2000 h, was maintained. Single housing commenced at the start of the behavioral experiment and continued throughout the experiment, which was conducted during the light period of the cycle. The animals used in this study were cared for in accordance with the guidelines of the National Institutes of Health Animal Care and Use Program and the NIDA-IRP Animal Program, and the present experiments were approved by the Austrian National Animal Experiment Ethics Committee.

### Place conditioning apparatus

Conditioning was conducted in a custom-built three chamber apparatus (63 cm wide × 33 cm deep × 30 cm high) made of plywood panels covered with plastic film. The middle (neutral) compartment (10 × 30 × 30 cm) had gray walls and a gray floor. The two conditioning chambers (25 × 30 × 30 cm) had either black walls with two vertical white stripes (5 × 30 cm) on each side and a stainless steel floor with 20 holes (diameter 0.5 cm) or black walls with five horizontal white stripes (3 × 25 cm) and a stainless steel floor with 15 slits (5 × 0.5 cm). Time spent in each compartment was measured with hand timers. If the summed duration for all three compartments was less than the total 900s of the test session, the missing time was distributed equally among the three chambers to avoid any bias. In 90% of the cases, the missing time varied from 3 s to maximum 15 s. This missing time was distributed equally among the three chambers, i.e., from 1 to 5 s in each chamber. After each rat, the apparatus was cleaned with a 70% camphorated ethanol solution.

### Place conditioning procedure

CPP was conducted as described previously (Fritz et al., 2011a). All experiments were performed by two individual experimenters using halogen white light (20 Watt) and radio-generated white noise.

### Acquisition of cocaine place preference

The conditioning procedure comprised a pretest session on day one, eight consecutive training days (alternate-day-design, one training session per day, a total of four training sessions each for cocaine or saline), and a CPP test on day 10. Pretest-, training-, and CPP test session lengths were of equal duration, i.e., 15 min = 900 s. The initially non-preferred chamber was subsequently paired with cocaine. Cocaine (hydrochloride salt, a gift from the National Institute on Drugs of Abuse to Gerald Zernig, corresponding to 15 mg/kg pure cocaine base in a volume of 1 ml/kg saline) or saline was injected intraperitoneally (i.p.) immediately before placing the rat into the closed dedicated chamber. The CPP test was performed 24 h after the last conditioning trial by placing the rat in the middle (neutral) compartment of the CPP apparatus and allowing it to move freely between the three compartments (*n* = 6).

### Acquisition of social interaction place preference

The conditioning procedure comprised a pretest session on day one, eight consecutive training days (alternate-day-design, one training session per day, a total of four training sessions each for social interaction or saline), and a CPP test on day 10 each 15 min. Pretest-, training-, and CPP test session lengths were of equal duration, i.e., 15 min = 900 s. The initially non-preferred chamber was subsequently paired with social interaction. If a compartment was paired with social interaction during CPP training, each rat received an i.p. injection of saline and was placed in the compartment to allow for social interaction with a conspecific of the same weight and gender (male) during the whole conditioning session. Each rat was assigned a different partner, which stayed the same for the whole duration of the experiment. Both animals remained singly housed. Observation indicated that only “agonistic” (i.e., “friendly”) social interaction, i.e., touching, crawling over and under, and allogrooming occurred, whereas “antagonistic” (i.e., threatening, boxing, fighting, biting etc.,) behavior was not observed during the training sessions (Kummer et al., 2012 under review). The CPP test was performed 24 h after the last conditioning trial by placing the rat in the middle (neutral) compartment of the CPP apparatus and allowing it to move freely between the three compartments (*n* = 5).

### Extinction of cocaine CPP: effect of social interaction

Only animals that had established cocaine CPP were used in the extinction and reinstatement experiments (35 of 38 rats used, 3 of 38 rats excluded). After cocaine CPP had been established, subjects were divided into two groups. One group (*n* = 17) received i.p. saline injections immediately before being put into the former cocaine-paired chamber as well as into the previously saline-paired chamber for one extinction session each (during a total of 2 consecutive days). The second group (*n* = 18) received an i.p. saline injection immediately before being placed into the previously cocaine-paired chamber for 15 min on 1 day but, in contrast to the previous group, was also given the opportunity to have social interaction in the previously saline-paired chamber with a conspecific on the other day. On the third day, animals were tested for CPP (15 min; T1). The three-day cycle of training-training-test was repeated three more times (T2, T3, T4).

### Cocaine-induced reinstatement of cocaine CPP: effect of social interaction

Twenty-four hours after the last extinction training, rats were administered a single i.p. cocaine injection in the previously cocaine-paired compartment. Another 24 h later, all rats were tested for CPP in a drug-free state (*n* = 13 per group).

### Immunohistochemistry

Zif268 immunohistochemistry was performed by an experimenter who was blind to treatment conditions as described previously (Fritz et al., 2011a).

Two hours after the start of the CPP test (for acquisition of cocaine CPP (*n* = 5) vs. social CPP (*n* = 5)-associated Zif268 expression) or the reinstatement test (for reinstatement of cocaine CPP with the presence (*n* = 8) or absence (*n* = 8) of alternate social interaction-associated Zif268 expression), rats were deeply anesthetized using isoflurane and intracardially perfused with 0.1 M phosphate buffered saline followed by 4% paraformaldehyde (PFA) dissolved in 0.1 M phosphate buffered saline (PBS; pH 7.4). Brains were then removed and postfixed in 4% PFA overnight, then stored in 30% sucrose at 4°C until the brain sank, and then at −80°C until sectioning. All serial brain sections (40 μm) were cut using a Cryostat (Leica). Sections were stored in an assorter buffer (Tris buffer 0.25 M, NaN3 10%) at 4°C until processed for immunolabeling.

Free-floating sections were washed in PBS 0.1 M and incubated for 30 min in 0.3% hydrogen peroxide/PBS 0.1 M. Then they were washed in PBS 0.1 M and incubated for 2 h in 0.3% Triton X-100 in PBS 0.1 M containing 3% bovine serum albumin (BSA). Subsequently, sections were incubated for 48 h at 4°C with the anti-Zif 268 rabbit polyclonal primary antibody (1: 3000 Santa Cruz Biotechnology sc-198, C-19, Santa Cruz) containing 0.3% Triton X-100, and 3% BSA in PBS 0.1 M. Sections were washed in PBS and incubated for 2 h in PBS containing biotinylated goat anti-rabbit antibody IgG (1:200, Vector Laboratories), 0.3% Triton X-100 and 3% BSA. Afterward, the tissue was given additional washes in PBS 0.1 M and incubated for 2 h in avidin biotinylated horseradish peroxidase complex (ABC Elite kit, Vector Laboratories) diluted in PBS 0.1 M. Then, sections were washed in PBS 0.1 M followed by a wash in 0.05 M Tris buffer (pH 7.6) for 10 min and they were incubated in 3,3-diaminobenzidine tetrahydrochloride containing 0.016% hydrogen peroxide for 3–8 min. This reaction was terminated by rinsing the tissue in 0.05 M Tris buffer then in PBS 0.1 M. Finally, sections were mounted onto gelatin-coated slides, dried and dehydrated before coverslipping.

**Figure 1:**
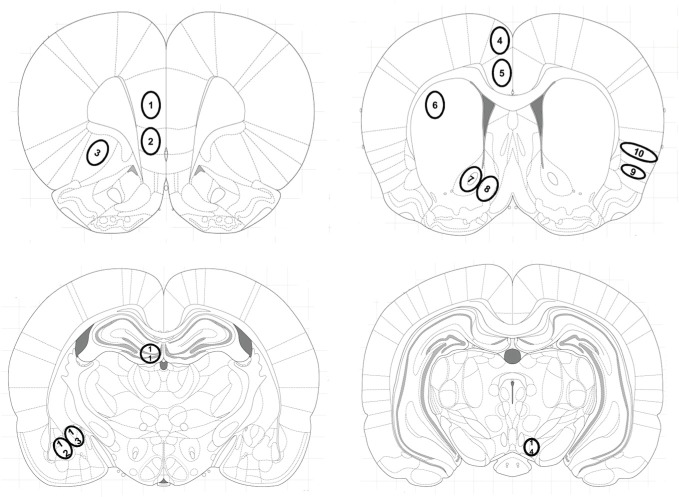
**Location of investigated brain areas**. Brain areas were identified according to the atlas of Paxinos and Watson (Paxinos and Watson, 2007): The (1) prelimbic (PrL), (2) infralimbic (IL) cortex and (3) the orbitofrontal cortex (LO) at Bregma 3 mm; the cingulate cortex areas (4) Cg1 and (5) Cg2, (6) dorsolateral striatum (caudate putamen, CPu), the (7) nucleus accumbens core (AcbC) and (8) shell (AcbSh) subregions, (9) the dorsal part of the agranular insular cortex (AID) and (10) the granular insular cortex (GI), at Bregma 1.08 mm; (11) the dentate gyrus (DG) of the hippocampus, (12) the basolateral (BLA) and (13) the central (Ce) amygdala at Bregma −2.64 mm and (14) the ventral tegmental area (VTA) at Bregma −5.04 mm.

Brain sections were scanned using a Zeiss optical microscope set at ×20 magnification equipped with a camera (Axioplan 2 Imaging) interfaced to a PC. Zif268-immunoreactive nuclei were counted by an observer who was blind to treatment conditions using Metamorph imaging software. Each rat contributed the averaged value of three sections per brain area to the group mean. Immunoreactivity was expressed as stained nuclei per mm^2^ of the respective brain regions identified according to the atlas of Paxinos and Watson (Figure 1) (Paxinos and Watson, 2007): The prelimbic (PrL) and infralimbic (IL) cortex, the lateral orbitofrontal cortex (LO), the anterior cingulated cortex areas Cg1 and Cg2, the dorsal striatum (caudate putamen, CPu), the nucleus accumbens core (AcbC) and shell (AcbSh) subregions, the granular insular cortex (GI), the dorsal part of the agranular insular cortex (AID), the dentate gyrus (DG) of the hippocampus, the central (Ce) and basolateral (BLA) amygdala, and the ventral tegmental area (VTA).

### Data analysis

The animals used in this study are different experimental groups on different time points within the same experiment. Therefore, statistical analyzes were allowed to be conducted between these experimental groups. All results are presented as group means ± SEM. All statistical tests were performed at a 0.05 level of significance with Statview software (SAS Institute Inc.). Behavioral results were analyzed using one-way, two-way repeated-measures or two-way analyses of variance (ANOVA). One-way repeated-measures ANOVA models always included the within-subjects factor compartment, two-factor repeated-measures ANOVA models included the factors compartment and time and two-way ANOVA included the factors compartment and treatment (saline vs. social interaction during extinction). Statistical significance of both main effects and interactions was tested. Moreover, *post-hoc* comparisons between individual factor levels (e.g., cocaine vs. saline) were performed using Tukey's test. Differences in Zif268 expression associated to acquisition of CPP **or** reinstatement of cocaine CPP in individual brain regions were performed using one-way ANOVA. *Post-hoc* comparison of individual treatments was done in the same manner as above. Differences in Zif268 expression associated to acquisition of cocaine CPP **vs**. reinstatement of cocaine CPP (cocaine CPP + saline extinction group) in individual brain regions were performed using Student's *t*-test.

Principal component analysis (PCA) was performed using varimax rotation of component matrices; factor loadings greater than 0.50 were retained as a factor. PCA was conducted with IBM SPSS.

## Results

### Acquisition of cocaine CPP and social interaction CPP

Animals acquired CPP to either social interaction alone or cocaine alone (Table 1). Repeated-measures ANOVA yielded a significant effect of the within-subject factor compartment, both for the social interaction group (*P* < 0.01, *F* = 17.5) and the cocaine group (*P* < 0.01, *F* = 13.8). *Post-hoc* comparison showed a significant preference, i.e., a longer time spent in the stimulus-associated compartment on CPP test day, for social interaction over saline in the social interaction group (*P* < 0.01) and a significant preference for cocaine over saline in the cocaine group (*P* < 0.01).

**Table 1:** **Acquisition of social interaction CPP and cocaine CPP**.

**Time in compartment mean (s) ± SEM**	**Drug or social interaction-paired compartment**	**Neutral compartment**	**Saline-paired compartment**
Cocaine CPP (*n* = 6)	387 ± 20^**^	277 ± 17	237 ± 13
Social interaction CPP (*n* = 5)	425 ± 31^**^	279 ± 15	241 ± 22

Shown is time spent in the cocaine or social interaction-, neutral, or saline-associated compartment. Shown are group means ± SEM

** P < 0.01 compared to the time spent in the saline chamber.

### Extinction and reinstatement of cocaine CPP: effect of social interaction on behavior

Cocaine CPP could be extinguished over four extinction cycles that were conducted over a period of 12 days. Two-way repeated-measures ANOVA with compartment and time as within-subject factors (CPP tests T1 to T4) performed during extinction revealed a significant compartment effect (previously cocaine-associated chamber vs. previously saline-paired chamber; *P* = 0.023, *F* = 6.35), a significant time effect (*P* = 0.002, *F* = 4.81) and a significant time-by-compartment interaction (*P* < 0.001, *F* = 13.2). For T1, a near-significant preference for the initially cocaine-associated compartment over the saline-associated compartment was found (*P* = 0.054, *F* = 4.33), whereas tests T2, T3 and T4 did not yield any significant preference for the cocaine-associated compartment any more (*P* > 0.1) (Table 2).

**Table 2:** **Extinction of cocaine CPP in the absence of social interaction**.

**Time in compartment mean (s) ± SEM**	**Drug-paired compartment**	**Neutral compartment**	**Saline-paired compartment**
Cocaine CPP (*n* = 17)	462 ± 17^**^	235 ± 9	203 ± 13
T1	377 ± 34	262 ± 14	262 ± 29
T2	316 ± 22	286 ± 13	280 ± 23
T3	316 ± 26	268 ± 16	316 ± 22
T4	299 ± 20	298 ± 16	303 ± 21

CPP, conditioned place preference test; T1–T4, conditioned place preference tests during extinction in absence of social interaction. Shown is the time spent in the initially cocaine- or saline- associated compartment and neutral compartment (group means ± SEM).

** P < 0.01 compared to the time spent in the saline chamber.

Social interaction in the previously saline-paired chamber during the first extinction cycle T1 eliminated the preference to cocaine in the subsequent CPP test. At T4, a preference for the social interaction-associated chamber developed showing a reversal of CPP from cocaine to social interaction (Table 3). Two-way repeated-measures ANOVA showed a non-significant compartment main effect (social interaction vs. saline, *P* > 0.1) and a non-significant time effect (*P* > 0.1) as a consequence of the reversal of CPP, but a highly significant compartment × time interaction (*P* < 0.001, *F* = 14.3). A significant preference of the saline-social interaction chamber over the initially cocaine-associated chamber was observed at T3 (*P* < 0.001, *F* = 19.7) and T4 (*P* < 0.001, *F* = 30.4), whereas no significant difference between the times spent in the respective chambers was seen at T1 (*P* > 0.05) and T2 (*P* > 0.05) (Table 3).

**Table 3:** **Extinction of cocaine CPP in the presence of social interaction**.

**Time in compartment mean (s) ± SEM**	**Drug-paired compartment**	**Neutral compartment**	**Saline + social interaction-paired compartment**
Cocaine CPP (*n* = 18)	420 ± 17^**^	247 ± 17	232 ± 10
T1	341 ± 37	245 ± 20	315 ± 29
T2	284 ± 26	273 ± 19	342 ± 25
T3	242 ± 20^**^	243 ± 14	416 ± 21
T4	244 ± 17^**^	248 ± 16	408 ± 16

CPP, conditioned place preference test; T1–T4, conditioned place preference tests during extinction in presence of social interaction made available in the initially saline-associated compartment. Shown is the time spent in the initially cocaine- or saline- associated compartment and neutral compartment (group means ± SEM).

** P < 0.01 compared to the time spent in the saline chamber.

If saline extinction of cocaine CPP was followed by one cocaine-chamber pairing session, i.e., reconditioning, fully extinguished cocaine CPP was reinstated (cocaine vs. saline compartment, *P* < 0.05) (Table 4). However, if social interaction was available in the previously saline-paired chamber during cocaine CPP extinction, reinstatement of cocaine CPP was not only fully prevented but preference for the social interaction-paired compartment remained (cocaine vs. social interaction compartment, *P* < 0.05) (Table 4) [two-way ANOVA, treatment effect (saline vs. social interaction), *P* > 0.05; compartment effect (cocaine vs. neutral vs. saline), *P* = 0.0208, *F* = 4.088; treatment × compartment interaction, *P* < 0.0001, *F* = 29.923].

**Table 4:** **Reinstatement of cocaine CPP in absence or presence of social interaction during extinction**.

**Compartment (s) ± SEM**	**Drug-paired compartment**	**Neutral compartment**	**Saline-paired or saline + social interaction-paired compartment**
Cocaine CPP after saline extinction (*n* = 13)	393 ± 17^*^	299 ± 12	208 ± 17
Cocaine CPP after counterconditioning to social interaction (*n* = 13)	242 ± 18^*^	325 ± 17	333 ± 25

Shown is time spent in the cocaine-, neutral, or saline- associated compartment. Shown are group means ± SEM.

* P < 0.05 compared to the time spent in the saline chamber or social interaction chamber.

### Zif268 expression associated to cocaine CPP and social interaction CPP acquisition: comparison with Zif268 expression associated to cocaine CPP reinstatement in presence or absence of social interaction during extinction

**Figure 2:**
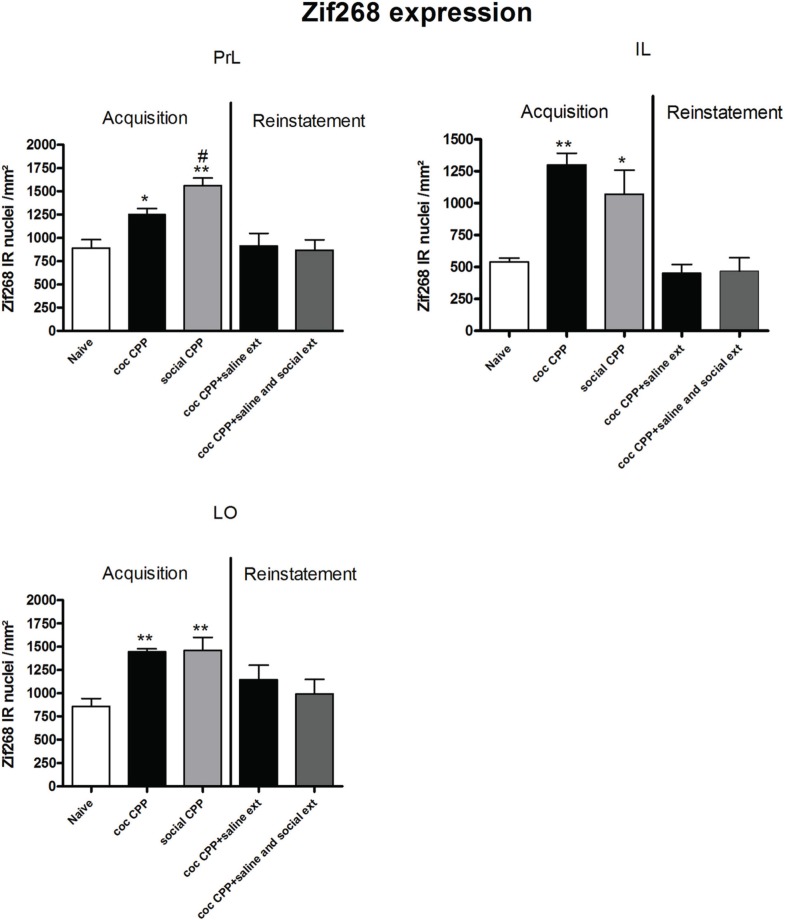
**Zif268 expression in the cortex after acquisition and reinstatement of CPP**. Zif268 expression was induced after cocaine CPP and social interaction CPP in the PrL, IL, and LO cortex. Social interaction CPP increased more Zif268 expression in the PrL than cocaine CPP. Shown are Zif268 positive immuno-reactive nuclei per mm^2^ of the respective brain area in naive rats, after acquisition of cocaine CPP (coc CPP) and after acquisition of social interaction CPP (social CPP) (left side of each graph of individual brain area). In addition, Zif268 expression was shown in rats that expressed cocaine CPP and underwent extinction in absence (coc CPP + saline ext) or presence of social interaction during extinction (coc CPP + saline and social ext) then reconditioned with cocaine in the previously cocaine-associated chamber (right side of each graph of individual brain area redrawn from Fritz et al., 2011a). Shown are group means ± SEM. ^*^ P < 0.05; ^**^ P < 0.01 compared to naive group; #*P* < 0.05 compared to coc CPP group. Brain area abbreviations follow the nomenclature of Paxinos and Watson (Paxinos and Watson, 2007).

**Figure 3:**
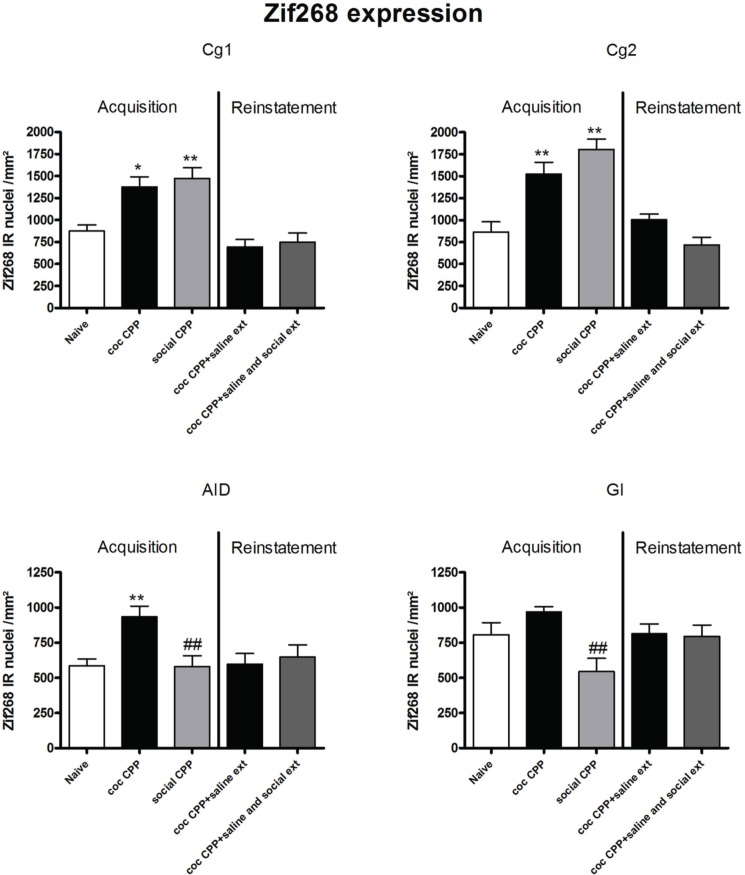
**Zif268 expression in the cortex after acquisition and reinstatement of CPP**. Zif268 expression was induced after cocaine CPP and social interaction CPP in the Cg1 and Cg2. Cocaine CPP increased Zif268 expression in the AID compared to naive rats while social CPP decreased Zif268 expression in the AID and the GI in comparison to cocaine CPP. Shown are Zif268 positive immuno-reactive nuclei per mm^2^ of the respective brain area in naive rats, after acquisition of cocaine CPP (coc CPP) and after acquisition of social interaction CPP (social CPP) (left side of each graph of individual brain area). In addition, Zif268 expression was shown in rats that expressed cocaine CPP and underwent extinction in absence (coc CPP + saline ext) or presence of social interaction during extinction (coc CPP + saline and social ext) then reconditioned with cocaine in the previously cocaine-associated chamber (right side of each graph of individual brain area redrawn from Fritz et al., 2011a). Shown are group means ± SEM. ^*^P < 0.05; ^**^P < 0.01 compared to naive group; ##*P* < 0.01 compared to coc CPP group. Brain area abbreviations follow the nomenclature of Paxinos and Watson (Paxinos and Watson, 2007).

**Figure 4:**
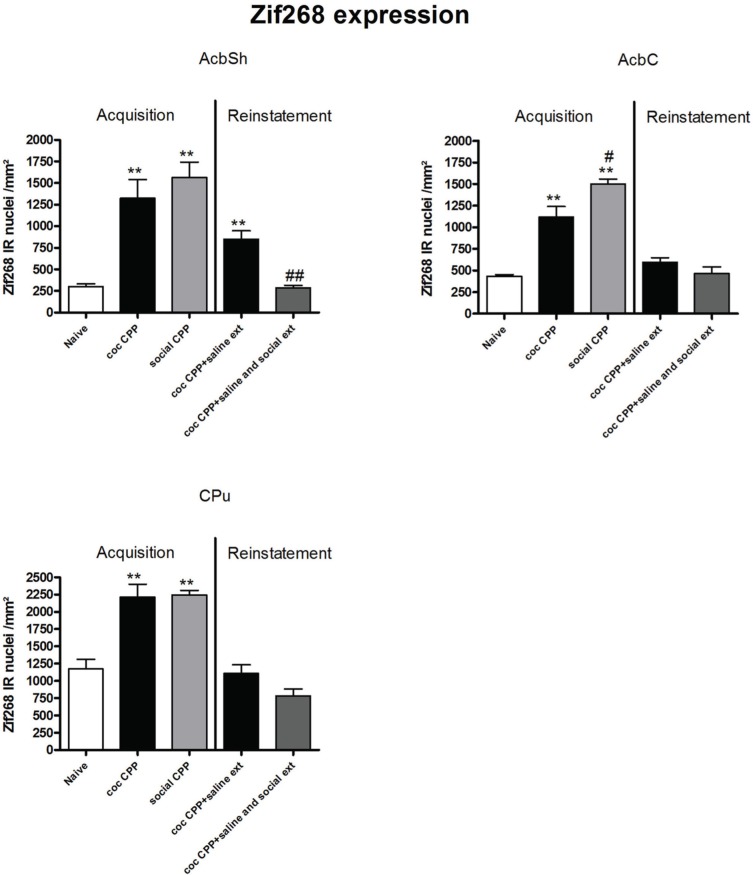
**Zif268 expression in the striatum after acquisition and reinstatement of CPP**. Zif268 expression was induced after cocaine CPP and social interaction CPP in the AcbSh, AcbC and the CPu. Social interaction CPP increased more Zif268 expression in the AcbC than cocaine CPP. In the AcbSh, social interaction reversed cocaine CPP reinstatement-associated Zif268 expression. Shown are Zif268 positive immuno-reactive nuclei per mm^2^ of the respective brain area in naive rats, after acquisition of cocaine CPP (coc CPP) and after acquisition of social interaction CPP (social CPP) (left side of each graph of individual brain area). In addition, Zif268 expression was shown in rats that expressed cocaine CPP and underwent extinction in absence (coc CPP + saline ext) or presence of social interaction during extinction (coc CPP + saline and social ext) then reconditioned with cocaine in the previously cocaine-associated chamber (right side of each graph of individual brain area redrawn from Fritz et al., 2011a). Shown are group means ± SEM. ^**^P < 0.01 compared to naive group; #*P* < 0.05 and ##*P* < 0.01 compared to coc CPP group for Zif268-associated acquisition of CPP or compared to coc CPP + social ext for Zif268-associated reinstatement of coc CPP. Brain area abbreviations follow the nomenclature of Paxinos and Watson (Paxinos and Watson, 2007).

**Figure 5:**
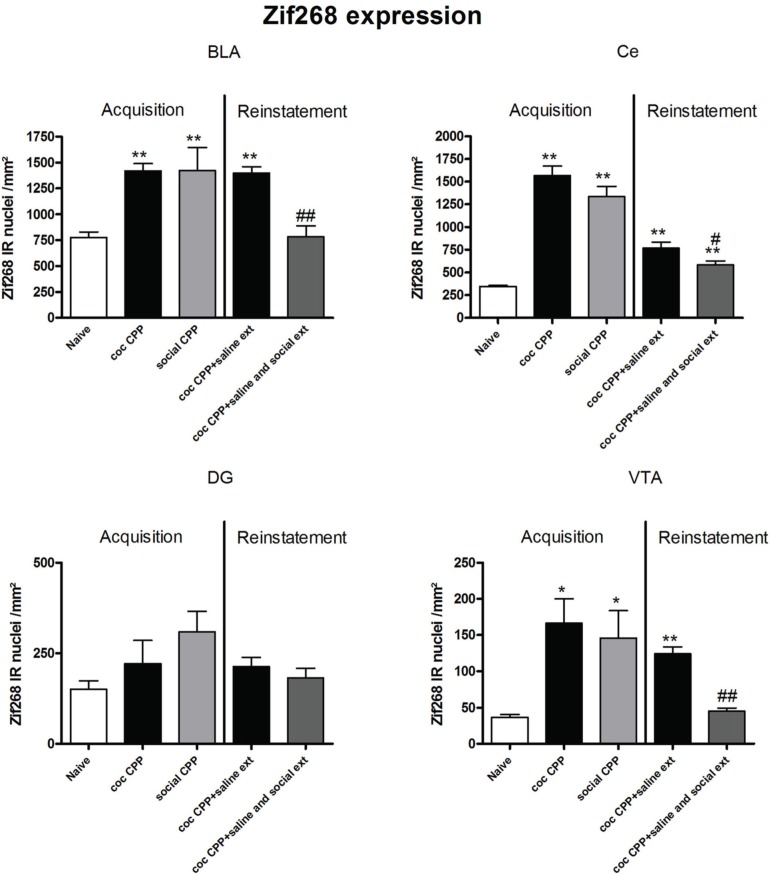
**Zif268 expression in the amygdala, hippocampus and ventral tegmental area after acquisition and reinstatement of CPP**. Zif268 expression was induced after cocaine CPP and social interaction CPP in the BLA, Ce and the VTA. In the BLA and the VTA, social interaction reversed cocaine CPP reinstatement-associated Zif268 expression. In the Ce, social interaction during extinction decreased cocaine CPP reinstatement-associated Zif268 expression. Shown are Zif268 positive immuno-reactive nuclei per mm^2^ of the respective brain area in naive rats, after acquisition of cocaine CPP (coc CPP) and after acquisition of social interaction CPP (social CPP) (left side of each graph of individual brain area). In addition, Zif268 expression was shown in rats that expressed cocaine CPP and underwent extinction in absence (coc CPP + saline ext) or presence of social interaction during extinction (coc CPP + saline and social ext) then reconditioned with cocaine in the previously cocaine-associated chamber (right side of each graph of individual brain area redrawn from Fritz et al., 2011a). Shown are group means ± SEM. ^*^P < 0.05; ^**^P < 0.01 compared to naive group; #*P* < 0.05 and ##*P* < 0.01 compared to coc CPP + social ext for Zif268-associated reinstatement of coc CPP. Brain area abbreviations follow the nomenclature of Paxinos and Watson (Paxinos and Watson, 2007).

Two hours after the cocaine CPP or the social interaction CPP test was performed, we investigated changes in activity levels of reward-related brain areas using Zif268 immunohistochemistry. Zif268 protein expression was different between treatments (naive *n* = 5, cocaine CPP *n* = 5, social interaction CPP *n* = 5) in all investigated areas (one way ANOVA, treatment effect, PrL: *P* = 0.0003, *F* = 17.540; IL: *P* = 0.0024, *F* = 10.342; LO: *P* = 0.001; *F* = 12.990; Cg1: *P* = 0.0033, *F* = 9.520; Cg2: *P* = 0.0005; *F* = 15.325; AID: *P* = 0.0042, *F* = 8.938; GI: *P* = 0.0075, *F* = 7.879; AcbS: *P* = 0.0003, *F* = 17.145; AcbC: *P* < 0.0001, *F* = 42.361; CPu: *P* = 0.0002, *F* = 19.128; BLA: *P* = 0.0012, *F* = 14.240; Ce: *P* < 0.0001, *F* = 54.131; VTA: *P* = 0.0187, *F* = 5.838) except in the DG (one way ANOVA, treatment effect, *P* > 0.05, *F* = 2.391). *Post-hoc* comparison showed that cocaine CPP increased Zif268 expression in the PrL (*P* < 0.05); IL (*P* < 0.01); LO (*P* < 0.01) (Figure 2); Cg1 (*P* < 0.05); Cg2 (*P* < 0.01); AID (*P* < 0.01) (Figure 3); AcbSh (*P* < 0.01); AcbC (*P* < 0.01); CPu (*P* < 0.01) (Figure 4); BLA (*P* < 0.01); Ce (*P* < 0.01) and the VTA (*P* < 0.05) (Figure 5) as compared to naive animals. In addition, social interaction CPP increased CPP expression in PrL (*P* < 0.01); IL (*P* < 0.05); LO (*P* < 0.01) (Figure 2); Cg1 (*P* < 0.01); Cg2 (*P* < 0.01) (Figure 3); AcbSh (*P* < 0.01); AcbC (*P* < 0.01); CPu (*P* < 0.01) (Figure 4); BLA (*P* < 0.01); Ce (*P* < 0.01) and the VTA (*P* < 0.05) (Figure 5) in comparison with naive rats. In comparison with social CPP rats, cocaine CPP produced significantly increased Zif268 expression in the AID (*P* < 0.01) and the GI (*P* < 0.01) (Figure 3) but significantly decreased Zif268 protein expression in the PrL (*P* < 0.05) (Figure 2) and the AcbC (*P* < 0.05) (Figure 4).

Zif268 expression, if tested 2 h after the cocaine reinstatement test performed in a cocaine-free state, i.e., 26 h after the last cocaine exposure, was significantly different between treatments (naive *n* = 5, cocaine CPP followed by saline extinction *n* = 8, and cocaine CPP followed by social interaction during saline extinction *n* = 8) in the following brain areas: AcbSh (one-way ANOVA, treatment effect: *P* < 0.01, *F* = 22.447), Ce (one-way ANOVA, treatment effect: *P* < 0.01, *F* = 16.160), BLA (one-way ANOVA, treatment effect: *P* < 0.01, *F* = 15.972) and VTA (one-way ANOVA, treatment effect: *P* < 0.01, *F* = 49.950). Cocaine CPP in the absence of alternate social interaction during extinction significantly increased Zif268 expression as compared to naive rats in the AcbSh (*P* < 0.01) (Figure 4), Ce (*P* < 0.01), BLA (*P* < 0.01), and the VTA (*P* < 0.01) (Figure 5). Social interaction during extinction of cocaine CPP completely reversed cocaine CPP-reinstatement-associated Zif268 expression to basal naive levels in the AcbSh (*P* < 0.01) (Figure 4), the BLA (*P* < 0.01), and the VTA (*P* < 0.01) (Figure 5). In addition, social interaction during extinction of cocaine CPP significantly reduced Zif268 expression compared to the saline group during extinction in the Ce (*P* < 0.05) (Figure 5).

Zif268 expression associated to cocaine CPP acquisition was found to be higher compared to Zif268 associated to reinstatement of cocaine CPP in absence of social interaction during extinction (cocaine CPP + saline ext group) in the IL (student's *t*-test, treatment effect, *P* < 0.01) (Figure 2); Cg1 (student's *t*-test, treatment effect, *P* < 0.01); Cg2 (student's *t*-test, treatment effect, *P* < 0.01); AID (student's *t*-test, treatment effect, *P* = 0.01) (Figure 3); AcbSh (student's *t*-test, treatment effect, *P* < 0.05); AcbC (student's *t*-test, treatment effect, *P* < 0.01); CPu (student's *t*-test, treatment effect, *P* < 0.01) (Figure 4) and the Ce (student's *t*-test, treatment effect, *P* < 0.05) (Figure 5).

### Principal component analysis of Zif268 expression associated with cocaine CPP and social interaction CPP

In each treatment group (i.e., cocaine CPP and social interaction CPP) four components were extracted using PCA, with one component being highly correlated with time spent in the stimulus-associated compartment (Table 5). In the cocaine CPP group, component 1 shows positive correlation with time spent in the cocaine-paired (time_in_coc) compartment (0.853), activation of brain regions Cg1 (0.974), AcbC (0.937), Cg2 (0.931), CPu (0.507), and negative correlation with activation of AID (−0.848). In the social interaction CPP group, component 2 shows positive correlation with time spent in the social interaction paired (time_in_int) compartment (0.918), activation of BLA (0.825), Cg2 (0.730), VTA (0.535), and negative correlation with activation of AcbSh (−0.936).

**Table 5:** **Principal component analysis (PCA) of brain region activation and behavior**.

**Rotated component matrix—cocaine CPP**					**Rotated component matrix—social interaction CPP**				
	**Component**					**Component**			
	**1**	**2**	**3**	**4**	**1**	**2**	**3**	**4**
PrL		0.839			PrL				0.900
IL		0.597		0.687	IL	0.875			
LO		0.539	0.832		LO	0.818			
Cg2	0.931				Cg2	0.506	0.730		
Cg1	0.974				Cg1	0.758		0.501	
CPu	0.507		0.649	0.529	CPu			0.954	
AcbSh		0.980			AcbSh		−0.936		
AcbC	0.937				AcbC				−0.854
DG			−0.899		DG	0.813			−0.521
Ce		0.927			Ce			0.992	
BLA			0.980		BLA		0.852	−0.504	
VTA				−0.961	VTA	0.825	0.535		
AID	−0.848				AID	0.969			
GI		0.569		0.547	GI	0.977			
Time_in_coc	0.853				Time_in_int		0.918		

Shown are factor loadings of brain regions and time spent in the stimulus associated compartment.

## Discussion

In this study, we aimed to investigate if the acquisition and expression of CPP for cocaine vs. social interaction produced different brain activation patterns. Our findings indicate that cocaine CPP and social interaction CPP activated almost the same brain regions. In some of these regions, the extent of activation was different after cocaine CPP and social interaction CPP. Indeed, we found that the granular insular cortex and the dorsal part of the agranular insular cortex were more activated after cocaine CPP, whereas the prelimbic cortex and the core subregion of the nucleus accumbens were more activated after social interaction CPP.

We have previously shown that CPP for 15 mg/kg i.p. cocaine and CPP for four 15-min episodes of social interaction were equally strong when rats were concurrently conditioned for place preference by pairing cocaine with one compartment and social interaction with the other (Fritz et al., 2011a,c). In the present study, we found that both conditioned stimuli were associated with an increase in Zif268 expression in the prelimbic, infralimbic, orbitofrontal, and the cingulate cortex. In addition, increased Zif268 expression was found in the striatum, the central and basolateral amygdala and the ventral tegmental area after cocaine CPP and social interaction CPP acquisition. Most of these areas had been previously shown to be associated with cocaine conditioned stimuli (Thomas et al., 2003; Miller and Marshall, 2005) and with social interaction stimuli (Salchner et al., 2004). The large overlap in brain regions involved in cocaine CPP and social interaction CPP acquisition suggests that even though the total number of Zif268-positive cells within many of the brain regions investigated were similar, Zif268 expression may have occurred in different cell populations (e.g., glutamatergic neurons vs. GABAergic interneurons) between each condition.

In comparison to social interaction CPP, the agranular insular cortex was only activated by cocaine-associated conditioned stimuli. Furthermore, we have found that Zif268 expression in the granular insular cortex was less pronounced after acquisition of social interaction CPP (Figure 3). Our results are in agreement with those of Contreras and colleagues who have found that amphetamine-seeking induced Fos expression in the insula. Insula inactivation in amphetamine-experienced rats prevented amphetamine seeking in a place preference task (Contreras et al., 2007). In a recent study, the same group has found that microinjection of anisomycin, a protein synthesis inhibitor, into the agranular insula immediately after the reactivation of the conditioned amphetamine/context memory produced a reversible but long-lasting loss of amphetamine-CPP and a decreased expression of zif268 in the insula (Contreras et al., 2012). These findings were interpreted as evidence that the insular cortex is involved in a context/drug effect association (Contreras et al., 2012). Furthermore, agranular insular cortex inactivation significantly attenuated cue-induced reinstatement of drug-seeking behavior (Di Pietro et al., 2006). Interestingly, it has been reported that damage of the insula leads to a profound disruption of addiction of cigarette smoking (Naqvi et al., 2007). Our results provide further evidence for agranular insular cortex activation during cocaine CPP acquisition/expression and suggest that the insular cortex was preferentially engaged by drug conditioned stimuli.

The rat prelimbic prefrontal cortex and nucleus accumbens core were reported to be critical for initiating cocaine seeking (Kalivas and O'Brien, 2008). It has also been shown that the inactivation of the PrL but not the IL cortex impaired drug-seeking behavior elicited by cocaine-associated stimuli (McLaughlin and See, 2003) and MDMA (“ecstasy”)-associated stimuli (Ball and Slane, 2012). We thus expected to find a more pronounced increase in Zif268 expression in the prelimbic cortex and in the core of the nucleus accumbens after cocaine CPP than after social interaction CPP. In the present study, we found that social interaction CPP activated the prelimbic cortex (Figure 2) and the nucleus accumbens core (Figure 4) more than cocaine CPP acquisition/expression did. In the context of CPP, whether the prelimbic cortex is required for cue-elicited drug seeking is more controversial: While Tzschentke and Schmidt reported that pre-conditioning excitotoxic lesions of the prelimbic cortex blocked the acquisition of cocaine CPP (Tzschentke and Schmidt, 1999), Neisewander and colleagues found that lesions of the prelimbic cortex did not affect the acquisition of cocaine CPP (Zavala et al., 2003). Furthermore, it has been shown that the PrL output to the nucleus accumbens core and the basolateral amygdala was attenuated during cocaine place preference expression (Miller and Marshall, 2005). Our findings suggest a role of the projection prelimbic cortex-core of the nucleus accumbens in the expression of non-drug associated conditioned stimuli.

We have recently shown that if rats were concurrently conditioned for place preference by pairing cocaine with one compartment and social interaction with the other, pre-acquisition lesioning of the accumbens core or the basolateral amygdala shifted the animals' preference toward social interaction (Fritz et al., 2011c). In the same study, we have also shown that pre-acquisition lesioning of the nucleus accumbens shell shifted the animals' preference toward cocaine preference in a mutually exclusive stimulus presentation during training cocaine vs. social interaction (Fritz et al., 2011c). These findings suggest a role of the nucleus accumbens core and the basolateral amygdala in mediating cocaine associated conditioned contextual stimuli and a role of the nucleus accumbens shell in mediating social interaction associated contextual stimuli. Accordingly, previous studies have also shown that lesions of the nucleus accumbens core but not the shell profoundly impaired the acquisition of cocaine-seeking behavior (Ito et al., 2004) and that inactivation of the nucleus accumbens core but not the shell abolished conditioned cue-induced reinstatement of cocaine seeking behavior (Fuchs et al., 2004). Also, (Fuchs et al., 2002) have found that pre-training lesions of the BLA disrupt acquisition of cocaine CPP and, See and colleagues have reported that inactivation of the BLA disrupted both the acquisition and expression of the conditioned reinforcing effects maintained by drug-paired stimuli (Kruzich and See, 2001). An interaction basolateral amygdala-nucleus accumbens core underlying stimulus-controlled cocaine seeking was reported by Di Ciano and Everitt (2004). In the context of CPP, Miller and Marshall suggested that the basolateral amygdala, rather than the prelimbic cortex, provides significant excitatory driving to the nucleus accumbens core during cue-elicited drug seeking (Miller and Marshall, 2005). We thus expected a higher activation of the basolateral amygadala and the nucleus accumbens core as mentioned above after acquisition/expression of CPP for cocaine than for social interaction, which was not the case. In the basolateral amygadala, we did not find such a difference (Figure 5). In corroboration of our previous lesion findings, the present study shows an increased Zif268 expression associated with social interaction CPP in the shell without, however, reaching statistical difference (Figure 4). These apparent partially contradictory results can be resolved by assuming that (1) only small and localized neuron ensembles within each brain region mediate conditioning to the respective stimuli (Koya et al., 2009) and (2) Zif268 expression allows the identification of brain regions that are activated, but it does not allow the determination of whether activating neurotransmitters or inhibiting neurotransmitters are released.

PCA showed that one of the four components extracted is correlated to the time spent in the stimulus-paired compartment. In the cocaine CPP group, Cg1, Cg2, CPu, AcbC, and AID were activated in concert and correlated to the time spent in the cocaine-associated compartment. We found that Cg1, Cg2, CPu, and AcbC were positively correlated but AID was negatively correlated with the time spent in cocaine compartment. A positive correlation between the agranular insula and cocaine seeking has been described previously (Kufahl et al., 2009). In this latter study, rats were first trained to press a lever then underwent extinction training, during which lever presses decreased. On the test day, rats received response-contingent cocaine cues that reinstated extinguished cocaine-seeking behavior. Therefore, the use of different paradigm could underlie the discrepancy in the direction of the correlation in the agranular insular cortex between our study and the study of Kufahl et al. (2009). Our findings suggest that connections between Cg1, Cg2, CPu, and AcbC regions play a role in cocaine seeking behavior. In the social interaction CPP group, Cg2, AcbSh, BLA, and VTA were activated in concert and correlated with the time spent in the social interaction-associated compartment. Cg2, BLA, and VTA were found to be positively correlated whereas AcbSh was negatively correlated with the time spent in social interaction compartment. In three of these regions, i.e., the AcbSh, BLA, and VTA, Zif268 expression that was associated with the reinstatement of cocaine CPP was almost completely abolished by a previous history of social interaction (Fritz et al., 2011a). Therefore, our findings suggest that connections between these regions could be involved in the expression of social interaction conditioned stimuli. Furthermore, the AcbC, AcbSh, and BLA have been reported to be implicated in cocaine associated conditioned stimuli or social interaction associated conditioned stimuli (Fritz et al., 2011c). Interestingly, the AcbC was previously shown to mediate cocaine associated conditioned stimuli (Fritz et al., 2011c) and; Zif268 in the AcbC at acquisition/expression of cocaine CPP time point was found to be positively correlated to the time spent in the cocaine compartment. Also, the AcbSh was previously shown to mediate social interaction associated conditioned stimuli (Fritz et al., 2011c) and; Zif268 in the AcbSh at acquisition/expression time point was found to be negatively correlated to the time spent in the social interaction compartment. We also expected to find a correlation between Zif268 expression in the BLA and the time spent in cocaine compartment in the present PCA analysis study as (1) our lesion study (Fritz et al., 2011c) has shown that the BLA mediates cocaine associated conditioned stimuli and (2) a positive correlation between the BLA and cocaine seeking behavior has been previously reported (Kufahl et al., 2009). However, we did not find a correlation between Zif268 expression in the BLA, i.e., BLA activation and the time spent in cocaine compartment. Possibly, the BLA could be implicated in mechanisms that are not directly correlated to the time spent in cocaine compartment but related to the expression of cocaine CPP such as learning and memory (e.g., component 3 extracted from the PCA analysis in the cocaine CPP group- Table 5). Indeed, three other components extracted from the PCA analysis also involve interactions between different regions in the brain during cocaine CPP or social interaction CPP acquisition (Table 5). These interactions could underlie various phenomenons/actions/pathways occurring in the brain during cocaine or social interaction CPP such as decision making, learning, etc.

When comparing acquisition and reinstatement of cocaine CPP-associated Zif268, we found that Zif268 protein was more strongly expressed after acquisition than after reinstatement of extinguished cocaine CPP in some brain regions such as the medial prefrontal cortex (infralimbic cortex and the cingulate cortex), the dorsal part of the agranular cortex, the striatum and the central amygdala. Several of these brain structures have been shown to be involved in extinction of a number of associative behaviors. For example, neuronal activity of the central nucleus of the amygdala has been reported to increase during conditioning and decrease during extinction training in an avoidance paradigm (Hernandez et al., 1990). Also, Morrow and colleagues have shown that acquisition, but not extinction of conditioned fear is associated with an increase in Fos expression in the medial prefrontal cortex (the prelimbic and the infralimbic cortex) (Morrow et al., 1999). The medial prefrontal cortex including the infralimbic cortex, seems to correspond to a limbic-related projection field receiving direct and indirect connections with limbic structures, such as the amygdala, olfactory structures, nucleus accumbens, hypothalamus, and hippocampus (Morrow et al., 1999). To emphasize, extinction is currently considered a form of learning that involves the formation of a new memory that suppresses behavioral responses to a learned stimulus (Bouton, 2002), although deletion of the previously acquired memory may also be involved (Myers and Davis, 2007). Therefore, in the context of CPP, structures the activation of which was decreased subsequent to extinction could be part of a circuitry that may be involved in the inhibition or the “unlearning” of the learned behavior.

In conclusion, the present study highlights the differential activation of some regions implicated in drug vs. non-drug acquisition preference. In the context of conditioned place preference, the insular cortex appears to be potently activated after drug conditioning learning while activation of the prelimbic cortex—nucleus accumbens core projection seems to be preferentially involved in the conditioning to the non-drug (“alternative”) stimuli that a “friendly” dyadic social interaction between weight-matched male rats can provide. Further work is necessary to characterize which neuron ensembles and which neurotransmitter systems mediate this aspect of drug- vs. social interaction reward.

## Authors contribution

Gerald Zernig, Rana El Rawas, Michael Fritz designed the experiments. Sabine Klement and Michael Fritz performed the behavioral study. Rana El Rawas performed the immunohistochemistry. Rana El Rawas analyzed the data. Kai K. Kummer performed PCA statistical analysis. Rana El Rawas and Kai K. Kummer wrote the paper. Gerald Zernig, Michael Fritz and Kai K. Kummer have critically reviewed the contents of the paper and provided instrumental suggestions. Alois Saria and Georg Dechant provided the infrastructure required to perform the experiments.

### Conflict of interest statement

The authors declare that the research was conducted in the absence of any commercial or financial relationships that could be construed as a potential conflict of interest.
